# Early prediction of 30-day mortality in patients with surgical wound infections following cardiothoracic surgery: Development and validation of the SWICS-30 score utilizing conventional logistic regression and artificial neural network

**DOI:** 10.1016/j.bjid.2025.104510

**Published:** 2025-02-21

**Authors:** Julio Alejandro Cedeno, Tania Mara Varejão Strabelli, Bruno Adler Maccagnan Pinheiro Besen, Rafael de Freitas Souza, Denise Blini Sierra, Leticia Rodrigues Goulart de Souza, Samuel Terra Gallafrio, Cely Saad Abboud, Diego Feriani, Rinaldo Focaccia Siciliano

**Affiliations:** a Instituto do Coração (InCor) do Hospital das Clínicas da Faculdade de Medicina, Universidade de São Paulo (HCFMUSP), São Paulo, SP, Brazil; b Instituto Dante Pazzanese de Cardiologia, São Paulo, Brazil; c Hospital das Clínicas da Faculdade de Medicina, Universidade de São Paulo (HCFMUSP), São Paulo, SP, Brazil; d Faculdade de Economia, Administração e Contabilidade de Ribeirão Preto (FEARPUSP), Universidade de São Paulo, SP, Brazil

**Keywords:** Wound infection, Cardiac surgery, Thoracic surgery, Mortality registries, Neural network model

## Abstract

**Introduction:**

We aimed to create and validate the 30-day prognostic score for mortality in patients with surgical wound infection (SWICS-30) after cardiothoracic surgery.

**Methods:**

This retrospective study enrolled patients with surgical wound infection following cardiothoracic surgery admitted to a Cardiologic Reference Center Hospital between January 2006 and January 2023. Clinical data and commonly used blood tests were analyzed at the time of diagnosis. An independent scoring system was developed through logistic regression analysis and validated using Artificial intelligence.

**Results:**

From 1713 patients evaluated (mean age of 60 years (18–89), 55 % female), 143 (8.4 %) experienced 30-day mortality. The SWICS-30 logistic regression score comprised the following variables: age over 65 years, undergoing valve heart surgery, combined coronary and valve heart surgery, heart transplantation, time from surgery to infection diagnosis exceeding 21 days, leukocyte count over 13,000/mm3, lymphocyte count below 1000/mm3, platelet count below 150,000/mm3, and creatinine level exceeding 1.5 mg/dL. These patients were stratified into low (2.7 %), moderate (14.2 %), and high (47.1 %) in-hospital mortality risk categories. Artificial intelligence confirmed accuracy at 90 %.

## Highlights

• Creation an score that can predict early mortality in patients with surgical wound infection after cardio thoracic surgery.
• Use an artificial intelligence (deep learning with artificial neural network) for validation.
• 8.3 % of mortality due to infection in patients with surgical wound infection after cardio thoracic surgery.

## Introduction

Infections after cardiothoracic surgery have been reported since the inception of these surgical procedures in the 1960s and 1970s. This type of infection significantly impacts morbidity and mortality rates and extends hospital length of stay, increasing costs for the healthcare system.^1^ The incidence of wound infections after cardiac surgery is estimated to range from 0.2 % to 8 %.^2^ It is reported that around 4 % of patients die after cardiac surgery while receiving hospital care.^3^ The longer their hospital stay, the higher the risk of mortality and the greater the cost of maintaining the patient's hospitalization.^4^ The risk of mortality in these patients is multifactorial, but one of the primary causes is surgical wound infection.^5^

The prognosis and likelihood of mortality after cardiothoracic surgery are influenced by several factors, including smoking, alcoholism, colonization by *Staphylococcus aureus*, hyperglycemia (diabetes), nutritional deficiency, obesity, renal dysfunction, and the type of cardiothoracic surgery perform.^6^, ^7^, ^8^, ^9^

Although the literature contains several scoring systems for assessing postoperative mortality, few are specifically focused on cardiac surgery, and even fewer address mortality related to Surgical Site Infections (SSIs) or sternotomy. Additionally, these scores often lack the ability to provide early predictions using easily accessible clinical and laboratory data across diverse care settings, such as outpatient clinics, hospital wards, emergency departments, and Intensive Care Units (ICUs). For example, tools like the EuroSCORE and ACS NSQIP, to predict patient prognosis, rely on clinical data such as comorbidities, preexisting dysfunctions (neurological, respiratory, arterial), active infections like endocarditis, surgical context (e.g., emergency surgeries), and specific cardiovascular variables such as angina, myocardial infarction, and ejection fraction. These factors, which are well-established predictors of higher mortality, are largely independent of surgical site infections.^10^

On the other hand, scores such as SOFA and APACHE II, serve as models for assessing disease severity based on organ dysfunction and complications in patients experiencing clinical deterioration, irrespective of the presence of an SSI. However, these tools are not applicable in outpatient settings. In contrast, the model proposed in this study (SWICS-30) is adapted for use across different levels of care in patients with SSIs following cardiothoracic surgery.

The SIS tool, designed to assess SSIs across various types of surgeries (e.g., abdominal, gynecological), demonstrates limited performance in the context of cardiothoracic surgery. Cardiothoracic procedures are classified as clean surgeries and are not directly comparable to abdominal or gynecological surgeries, where the microbiota and associated infection risks differ significantly, requiring distinct prophylactic measures and resulting in higher infection risks relative to cardiothoracic procedures.

Introducing a tool capable of predicting the risk of early mortality in patients with Surgical Wound Infections (SWI) following cardiothoracic surgery should provide valuable support for medical decision-making processes. This study aimed to develop and validate the SWICS-30 score (Surgical Wound Infection after Cardiothoracic Surgery ‒ 30-day mortality risk score) for early mortality prediction in patients with wound infections after cardiothoracic surgery. Due to the fact that it was an early score, which would mean the first hours, microbiological variables were not included, due to the growth time of cultures, which generally takes 24‒28 h.

## Methods

### Type of study and patient selection

This retrospective study examined patients with surgical wound infections after cardiothoracic surgery who were admitted to the Cardiologic Reference Center Hospital (Instituto do Coração ‒ InCor), University of São Paulo, Brazil, between January 2006 and January 2023. Patient selection was conducted prospectively as part of routine surveillance activities by the Hospital Infection Control Department.

This department monitors wound infections in hospitalized patients and actively investigates outpatient infections. Infections were categorized based on definitions established by the Centers for Disease Control and Prevention (CDC)^11^ and classified as superficial, deep, or organ space (mediastinitis), with osteomyelitis not differentiated in this cohort. The construction of the scoring system adhered to the “Guide for presenting clinical prediction models for use in clinical settings”, and the results were reported following the “Transparent Reporting of a multivariable prediction model for Individual Prognosis or Diagnosis (TRIPOD) statement”.^12^

### Inclusion and exclusion criteria

The study included patients diagnosed with surgical wound infections (as per Centers for Disease Control and Prevention guidelines)^11^ following various types of cardiothoracic surgeries, such as Coronary Artery Bypass Graft Surgery, Coronary Artery Bypass Graft Surgery combined with valve procedures, valve surgery, aorta/vascular surgery, heart transplant, congenital heart surgery, and general cardiac surgery. Patients under 18-years old were excluded from the study.

### Outcomes

The primary outcome was the occurrence of 30-day death from all causes after diagnosis of wound infections after cardiothoracic surgery. All participants were followed for at least 30-days following their diagnosis of wound infection.

### Data collection

The predictor variables studied were collected by reviewing medical records on the day of infection diagnosis, such as age, body mass index, presence of fever, hemoglobin, leukocyte, lymphocyte, platelet, C-reactive protein, and creatinine levels. Chest tomography reports performed up to 7-days after the diagnosis of infection were studied, and the following characteristics were evaluated: bilateral pleural effusion, mediastinal collection, bone resorption, bone misalignment, and bone diastase. The primary surgery type and the time elapsed between the diagnosis of infection and the surgical cleaning approach were also collected.

### Statistical analysis and scoring system

Two models were used in this study. Establishing binary logistic regression allowed researchers to study the marginal impacts of the predictor variables on the phenomenon studied (i.e., ORs) and propose the SWICS-30 score. After this, a Deep Learning (DL) Artificial Neural Network (ANN) was used to validate the results of the SWICS-30 score further.

We stratified the descriptive statistics based on in-hospital mortality. We presented them as means and standard deviations or medians and 25th/75th percentiles for continuous variables, as appropriate for their distribution, and as counts and percentages for categorical variables. The analysis involved *t*-tests or Wilcoxon rank-sum tests for continuous variables, as appropriate, and the Chi-Square test for categorical variables.

### Logistic regression model derivation

Predictor variables were selected after thorough review of existing literature of surgical wound infections after cardiothoracic surgery. For multivariable analysis, the selected variables included age ≥ 65-years, surgical group such as valve group, transplant group, coronary-valve group, time between surgery and diagnostic of infection, leukocytes > 13.000 mm^3^, lymphocytes 〈 1.000 mm^3^, platelets < 150.000 mm^3^, creatinine 〉 1.5 mg/dL.

These variables were integrated into an initial model, and a multivariable logistic regression was performed, with a purposeful backward selection of variables to create the final model based on their contribution to the model explainability by likelihood ratio tests and information criteria. Our objective was to develop the most parsimonious model. Afterwards, we developed a scoring system, assigning points based on logistic regression coefficients for each variable. Finally, patients were categorized into three distinct risk levels of mortality by analyzing the numerical and graphical distribution of the risk of this outcome and testing the optimal cutoff point for group differentiation based on the clinical decision-making required at the bedside. The score was given using the logarithmic coefficients from the logistic regression following the Guide for presenting clinical prediction models for use in clinical settings.

Apparent validation was conducted by analyzing the Area Under the Receiver Operating Characteristic (AUROC) curve and a calibration belt. Bootstrap internal validation was performed with 500 bootstrap resamples for AUROC, calibration-in-the-large, calibration slope and a calibration plot.

All tests were 2-sided, and a p-value < 0.05 was considered statistically significant. No adjustment for multiplicity was performed. The analysis was performed using StataSE® software (StataCorp, College Station, Texas), version 18.0.

### Deep learning artificial neural networks

To enhance the robustness of the study, a DL model was suggested that made use of the SWICS-30 scores recommended by the logistic estimation discussed earlier. The established DL model was a multi-layer feed-forward artificial neural network, estimated using the h2o package, version 3.44.0.3, of the R computer language core team.

The DL model was trained with 70 % of the sample using a cross-validation technique with 10k-folds and misclassification as a stopping metric. The algorithm was trained using stochastic gradient descent and backpropagation criteria.^13^

For the input layer of the deep learning estimation, the same predictor variables utilized in the binary logistic model were taken into account. However, the variables age, time between surgery and diagnosis of infection, leukocytes, lymphocytes, platelets, and creatinine were considered in their original metric forms. The hyperparameters of the DL model were established using grid simulations following a random discrete strategy.^14^^,^^15^

The aforementioned grid simulations were also used to establish an ideal number of three hidden layers for the ANN, with 600 neurons each, and helped to choose the activation functions for each layer of the model. The rectifier function was used to calculate the interaction between neurons in the hidden layers.

For both the hidden and output layers, we opted for lasso regression (L1) and ridge regression (L2) regularizations^16^^,^^17^ with weights of 0.00001 and 0.001, respectively. The aim was to add a penalty term to the selected loss function,^18^ namely, cross entropy. All the choices made helped minimize the log loss metric, both for the training and the validation samples, to mitigate the possibility of overfitting the model.^19^

Finally, given that, the proposed SWICS-30 scores are polytomous categorical variables (i.e., Low, middle and High), a SoftMax activation function was considered for the output layer.

### Ethical considerations

This study was approved by the Ethics and Research Committee of the Instituto do Coração (InCor), University of São Paulo, Brazil. Heart Institute of the Faculty of Medicine of the University of São Paulo and registered under SDC 3695|11|113 and CAAE 31,593,814.8.0000.0068. The requirement for informed consent was waived due to the retrospective nature of the study.

## Results

**Fig. 1:**
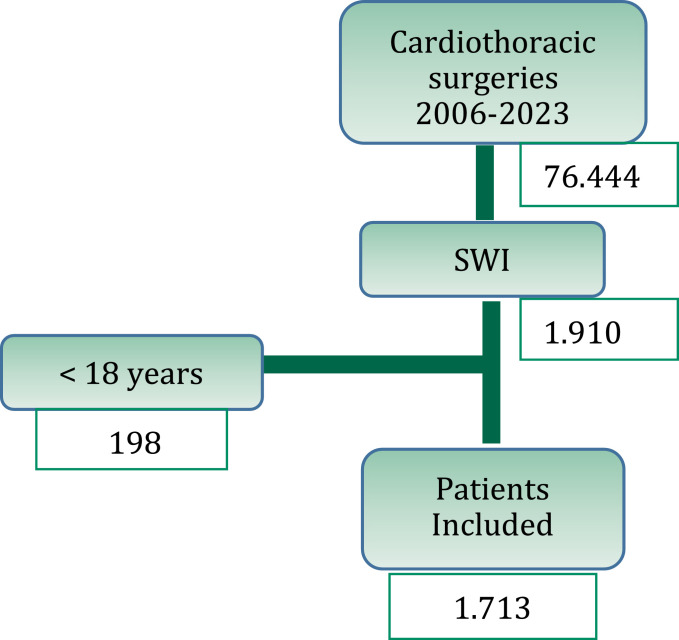
Patients involved and selection.

Between 2006 and 2023, a total of 76,444 cardiothoracic surgeries were performed, resulting in 1911 episodes of surgical wound infections (2.5 %‒1911 %/76,444). Out of these, 198 cases were excluded as they involved patients under the age of 18. Therefore, 1713 cases were analyzed (Fig. 1).

### Patient characteristics

In the present study, there was a female predominance of 55 %. The mean age of participants was 60 years (interquartile range: 18‒89). The baseline characteristics of the participants, including demographics, risk factors, signs and symptoms, laboratory tests, microorganisms, and surgical group, are described in Table 1.

**Table 1:** Characterization of patients with surgical wound infection and death.

**Variables**	**Total** **(*n* = 1713)**	**Survivors** **(*n* = 1570)**	**Non survivor** **(*n* = 143)**	**p-value**
Demography				
Female sex	936 (55 %)	863 (55 %)	73 (51 %)	0.38
Mean age (SD)	60.4 (13.4)	59.9 (13.4)	65.1 (12.8)	<0.001
BMI, median (IQR) (*n* = 1457)	27.3 (24.1, 31.2)	27.3 (24.2, 31.2) (*n* = 1339)	26.5 (23.4, 31.1) (*n* = 118)	0.19
**Clinical characteristics**	**n (%)**			
**Surgery group (*n* = 1683)**				
Valve	389 (22.7 %)	343 (21.8 %)	46 (32.2 %)	0,007
Coronary	919 (53.6 %)	871 (55.5 %)	48 (33.6 %)	<0.001
Coronary-valve	39 (2.3 %)	30 (1.9 %)	9 (6.3 %)	0.004
Congenital	67 (3.9 %)	66 (4.2 %)	1 (0.7 %)	0.039
Cardiac general surgery	83 (4.8 %)	71 (4.5 %)	12 (8.3 %)	0.093
Heart and lung transplant	49 (2.9 %)	38 (2.4 %)	11 (7.7 %)	0.002
Aorta/vascular	137 (7.9 %)	122 (7.7 %)	15 (10.5 %)	0.44
**Comorbidities**				
Diabetes	517 (30.2 %)	487 (31.0 %)	30 (21.0 %)	0.013
**Signs and symptoms**				
Fever (*n* = 1529)	262 (17.1 %)	235 (16.9 %)	27 (19.1 %)	0.48
Time between surgery to diagnostic, median (IQR)		16.4 (10, 26.2) (*n* = 1570)	21 (10.7, 28) (*n* = 143)	0.074
**Blood tests**				
Hemoglobin, mean (SD)	9.7 (1.7)	9.8(1.7) (*n* = 1444)	9.1(1.6) (*n* = 142)	<0.001
Leukocyte, median (IQR)	10,420 (8010, 13,680)	10,300 (7930, 13,490) (*n* = 1436)	12,370 (9360, 17,820) (*n* = 142)	<0.001
Lymphocyte, median (IQR)	1525 (1076,2138)	1551.5 (1106, 2171) (*n* = 1550)	1161 (689, 1697) (*n* = 140)	<0.001
Creatinine, median (IQR)	1.1 (0.9, 1.6)	1.1 (0.9, 1.5) (*n* = 1422)	1.9 (1.2, 2.9) (*n* = 141)	<0.001
C reactive protein, median (IQR)	95.4 (48.6, 162.4)	93.8 (46, 157.2) (*n* = 1385)	123.4 (61.9, 201.2) (*n* = 130)	<0.001
**Imaging (*n* = 1509)**				
Bilateral pleural effusion	382 (25.3 %)	356 (25.5 %)	26 (22.6 %)	0.58
Mediastinal collection	275(18.2 %)	256 (18.3 %)	19 (16.5 %)	0.71
Bone resorption	106 (7 %)	103 (7.4 %)	3 (2.6 %)	0.056
Bone misalignment	30 (1.9 %)	27 (1.9 %)	3 (2.6 %)	0.49
Bone diastase	19 (1.3 %)	16 (1.1 %)	3 (2.6 %)	0.17

The 30-days overall mortality rate was 8.3 % (143/1713). The comorbidity highlighted was diabetes mellitus in 517 (30 %) cases. Coronary surgery was the largest surgical group with 919 patients (53 %), followed by the valve group with 389 patients (23 %) and the aorta/vascular group with 137 patients (7.9 %).

The classification of surgical wound infections was as follows: 660 patients (38.5 %) had superficial infections, 770 patients (44.4 %) had deep infections, and 179 cases (10.4 %) had mediastinitis.

*Staphylococcus* spp. coagulase-negative and *Staphylococcus aureus* were the most commonly observed microorganisms, with 455 cases (26 %) and 343 cases (20 %) respectively. Gram-Negative Bacilli (BGN) were also frequently identified, accounting for 465 cases (27 %), while fungi were detected in 133 cases (7.7 %). *Enterococcus spp*. were identified in 77 cases (4.5 %), and *Streptococcus spp*. were found in 13 cases (0.7 %). It is important to highlight that 14 % of cases had negative culture results or were not collected due to the presence of superficial infections with poor secretion. Surgical cleaning of the wound was indicated in 934 instances (55 %) and occurred on average 21 days (± 35-days) after diagnosis of the infection. Among the 934 individuals who underwent surgical intervention, 696 (75 %) of them utilized negative pressure wound therapy.

### Model derivation

Results of the final logistic regression model are described in Table 2. The variables and their respective scores are detailed in Table 3.

**Table 2:** Factors associated with mortality in patients diagnosed with surgical wound infection after cardiothoracic surgery - multivariate logistic regression of the score.

	**Odds Ratio**	**95 % Conf. interval**	**Std. Err.**	**p-value**
Age > 65 years	1.86	1.27‒2.82	3.85	0.002
Valve surgery	1.69	1.06‒2.71	4.05	0.026
Coronary-valve surgery	3.84	1.6‒9.23	1.72	0.003
Heart and lung transplant	2.53	1.09‒5.87	1.09	0.031
Time between surgery and diagnostic of infection (21 days)	1.91	1.29‒2.84	3.86	0.001
Leukocytes > 13,000	2.11	1.41‒3.15	4.32	<0.001
Lymphocytes < 1000	1.65	1.08‒2.5	3.51	0.019
Platelets < 150,000	2.54	1.60‒4.04	6.01	<0.001
Creatinine > 1.5	7.61	4.69‒12.33	1.87	<0.001

**Table 3:** Predictive score for mortality due to surgical wound infection after cardiothoracic surgery. The SWICS‒30 score.

**Variable**	**Points**
Age > 65 years	+1
Valve Group	+1
Transplant Group	+2
Coronary-valve Group	+3
Time between surgery and diagnostic of infection (21‒Days)	+1
Leukocytes > 13.000/mm^3^	+1
Lymphocytes < 1000/mm^3^	+1
Platelets < 150.000/mm^3^	+2
Creatinine 1.3–2.0 mg/dL	+2
Creatinine > 2.0 mg/dL	+4

The risk groups were established as follows: low risk (1 to 5-points), moderate risk (6 to 8-points) and high risk (≥ 8-points) of death, as shown in Table 4. The incidence of hospital death was 2.7 %, 14.2 % and 47.1 % for each risk group, respectively.

**Table 4:** Risk stratification of mortality due to surgical wound infection after cardiothoracic surgery according to the SWICS‒30 score.

**Risk group**	**Number of patients (%)**	**Risk of 30‒day mortality (%)**
Low (1‒5)	958 (62 %)	26 (2.7 %)
Middle (6‒8)	500 (32 %)	71 (14.2 %)
High (> 8)	87 (6 %)	41 (47.1 %)
Total	1545	138

### Model apparent validation

**Fig. 2:**
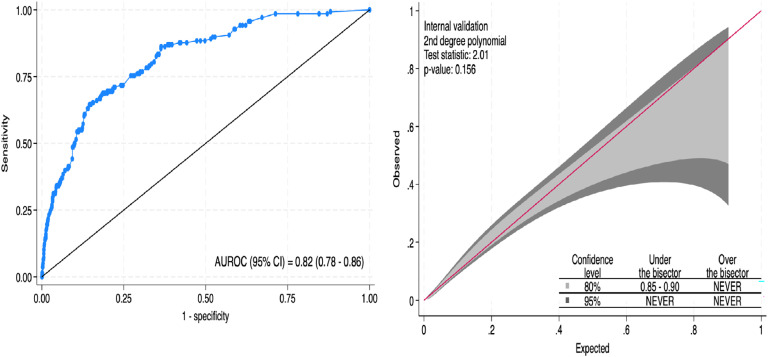
Apparent validation of the logistic regression model. (A) An Area under the receiving operator characteristic curve. (B) Calibration belt of the model.

The SWICS-30 score presented an area under the ROC curve of 0.82 (95 % CI 0.78‒0.86), as shown in Fig. 2A. Model calibration, evaluating the alignment between the predicted probabilities and the actual observed results, was considered adequate. The calibration belt in Fig. 2B visually illustrates this agreement.

### Internal validation

**Fig. 3:**
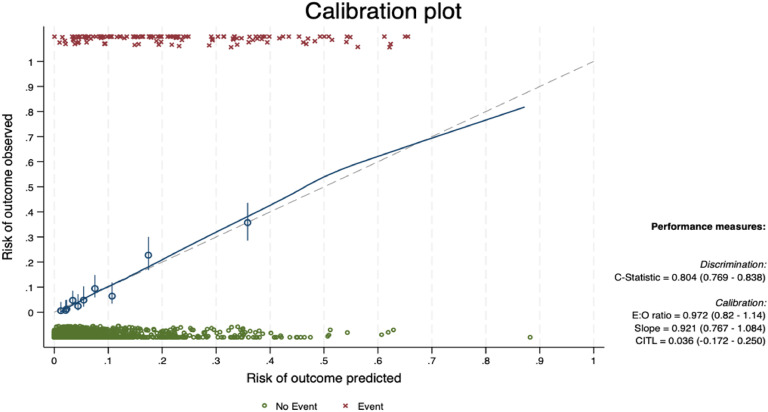
Calibration plot after bootstrapping internal validation.

The bootstrap internal validation with 500 replicates yielded an AUROC of 0.804. Calibration-in-the-large was 0.036 and calibration slope was 0.921. The calibration plot after bootstrapping internal validation is presented in Fig. 3.

### AI validation

The DL model was used to validate the SWICS-30 score, considering the Low, Middle and High labels (Table 4). To this end, patients who had missing values among the predictor variables, making it impossible to calculate their SWICS-30 score, were disregarded. It is important to mention that, a priori, all the variables described in Table 1 were considered in the modeling, but dichotomous predictor variables with few events (i.e., <30) were also disregarded. The study variables originally presented as quantitative variables (Table 1) were considered in their observed format, that is, as continuous variables. Therefore, of the 1713 individuals in the initial sample; 1554 were considered for ANN modeling. The validation results are presented in Table 5.

**Table 5:** Validation of SWICS‒30 using ANN.

**Observed SWICS‒30**	**Predicted SWICS‒30**		
	**Low**	**Meddle**	**High**
Low	891	75	0
Meddle	57	438	6
High	0	18	69

If we consider the sum of the elements on the main diagonal of Table 5 and divide them by the sample considered by the ANN, we see that the accuracy is approximately 90 %.

However, unlike a logistic regression model, ANNs cannot be summarized as a single mathematical equation, making it impossible to calculate the ORs of the predictor variables for the phenomenon studied. Nevertheless, there is the possibility of an approximation given by the so-called Partial Dependence Plots (PDP).^20^

**Fig. 4:**
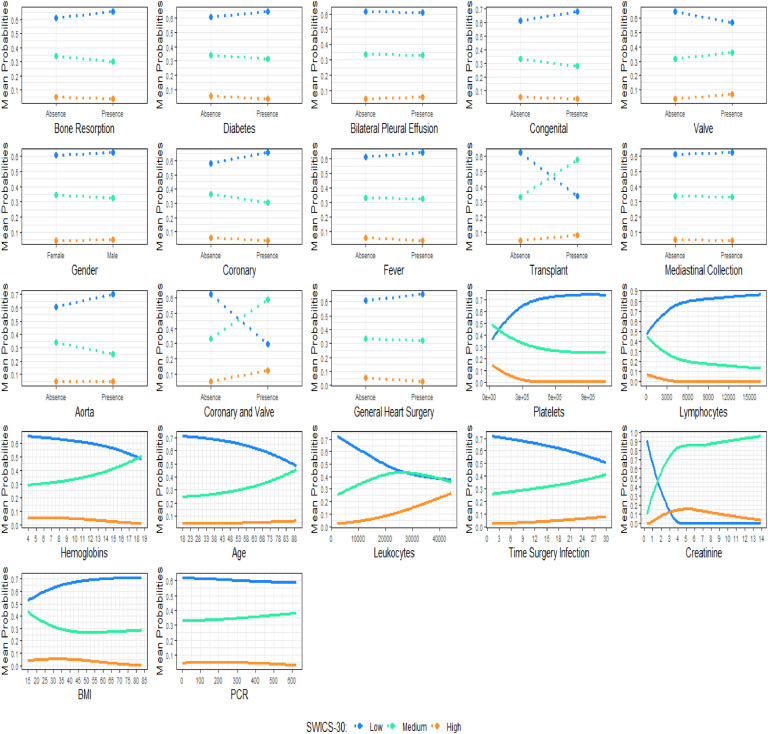
Partial dependent plots of the ANN model.

The PDPs propose visualizing the relationship between a given set of explanatory variables and the dependent variable, considering their average impact on the phenomenon studied.^21^ It must be said that the PDPs assume independence between a given predictor variable and the rest of them in relation to the phenomenon. Fig. 4 shows the PDP for each predictor variable in this study.

Fig. 4 shows the behavior of the variables present in the base in relation to the SWICS-30 score, assuming Low, Middle and High categories, as shown in Table 4.

When analyzing the dichotomous explanatory variables, the results presented in Fig. 4 suggest that patients who underwent coronary-valve surgery, valve surgery, heart transplant, or who presented bilateral pleural effusion, are more likely to be categorized by the SWICS-30 score as middle or high-risk patients. In fact, with the exception of the bilateral pleural effusion variable, the presence of the aforementioned variables leads to a sharp drop in the probability of a patient being considered low risk in the SWICS-30 score.

The outcomes of the study's metric variables, as depicted in Fig. 4, suggest that an increase in the time between surgery and the diagnosis of wound infection, as well as a rise in the age variable, leads to a higher likelihood of a patient being categorized as middle or high-risk in the SWICS-30 score.

The researchers emphasize once again that the interpretations of the PDP are independent of the joint behavior of the other predictors in the phenomenon studied.

## Discussion

This investigation introduces the SWICS-30 score, a recently developed and validated system designed to predict hospital mortality within a large cohort of patients with surgical site infections after cardiothoracic surgery. Utilizing early clinical and laboratory data exclusively, the system employs two models, without considering blood culture or treatment modalities, to assess the risk of hospital mortality early. Consequently, this score holds the potential for aiding clinicians in promptly identifying patients with a lower likelihood of survival and directing healthcare resources, such as transferring them to tertiary centers capable of performing debridement surgeries.

Our literature review found no other scoring system capable of accurately predicting mortality risks associated with surgical wound infections in patients undergoing cardiothoracic surgery. While several recent studies have assessed predictors for mortality in patients with wound infections following cardiovascular surgery, they often examined relatively small cohorts ranging from 15 to 1500 participants, albeit with similar surgical groups as our study (e.g., Coronary Artery Bypass Graft Surgery, valve surgery, combined surgery, and aortic surgery).^7^, ^8^, ^9^^,^^22^, ^23^, ^24^, ^25^

The present study revealed that the 30-day mortality rate due to surgical wound infections after cardiothoracic surgery was 8.3 %. The reported mortality rates for patients with surgical wound infections after cardiac or cardiothoracic surgery have been found to differ across various studies. As an instance, El-Din showed a mortality rate of 22.6 % for deep infections,^9^ while Filsoufi documented a 14 % mortality rate for the same type of infections,^22^ Kotnis-Gaska also disclosed a mortality rate of 21 %,^24^ Moinipoor detailed a rate of 10.9 %,^25^ Kaspersen reported a rate of 8 %^7^ and Abboud revealed a rate of 10 % only for mediastinitis cases.^26^

According to this study, age 65 and above emerged as a significant risk factor for mortality, which is consistent with the findings of other studies that have used age as a risk factor for mortality.^22^^,^^27^ These studies have found that patients with a higher risk of mortality due to surgical wound infection (mediastinitis) typically fall within the age range of 64 to 76 years.

The SWICS-30 score revealed that the coronary-valve group had the highest risk of mortality, followed by the heart transplant and valve group. These findings were consistent with the results of previous studies conducted by Filsoufi, Kaspersen, and Banjanovic, in which the coronary-valve group had the highest mortality rate.^7^^,^^22^^,^^23^ This can be attributed to the complexity and extensive nature of the procedure. On the other hand, another recently published study failed to demonstrate any statistically significant variation between the surgical groups for coronary, valve, aorta, and combined procedures.^8^

The current investigation uncovered that a patient diagnosed with surgical wound infections beyond 21-days after cardiothoracic surgery had a higher risk of death. In two other studies, the median time was lower (12-days and 16-days), but this variable was not associated with increased risk of mortality.^8^^,^^9^ The delay in diagnosis, which might be caused by mild local symptoms or a lack of attention from either physicians or patients, can lead to more severe consequences for these individuals. The results of some laboratory tests at the time of infection were important risk factors for mortality. We found that leukocytosis, thrombocytopenia, lymphopenia and creatinine >1.5 mg/dL were associated with high mortality rates. Patients who were already on dialysis might have had extremely high creatinine levels upon admission, and this did not serve as a prognostic marker, in contrast to the infection-related elevation in creatinine seen in numerous patients. Krasivskyi also observed that an increase in creatinine was associated with increased mortality.^10^ Unfortunately, we were unable to identify any other studies that assessed the prognostic value of the complete blood count or C-reactive protein for wound infections following cardiothoracic surgery.

In recent decades, some scoring systems have been developed to estimate the risk of surgical wound infections after cardiac surgery, such as the Barts Score,^1^ National Nosocomial Infection Score, Australian Clinical Risk Index (ACRI),^28^ and Brompton and Harefield Infection Score (BHIS).^29^ Each of these tools has its unique features. For instance, the ACRI and BHIS are limited to assessing infections in patients undergoing Coronary Artery Bypass Grafting (CABG), while the Barts Score evaluates both CABG patients and those undergoing valve surgery. However, these studies do not evaluate mortality.

The key advantage of our study lies in its comprehensive approach, which encompasses all types of cardiac surgeries and includes patients across varying levels of clinical severity ‒ ranging from outpatient settings to inpatient wards, emergency departments, and ICUs. This broader scope differentiates our study from others, which often focus exclusively on ICU patients, where high mortality rates are already well-documented.

In this research, two mathematical models were employed, and their accuracy varied based on the structure of each method. ANN often involves a larger number of variables and their intricate interrelations, although they cannot specifically account for the weight of each one in the relationship. The deep learning study integrates tomographic, clinical, and laboratory characteristics. For example, the following three variables were identified as having greater relative importance in this analysis, despite not being included in the logistic regression model (Fig. 4): fever at diagnosis, which indicates a systemic inflammatory response due to infection, similar to leukocytosis and suggests a more severe illness; and bone resorption observed in chest tomography, which was not studied by other authors and may be a protective factor associated with more benign cases of osteomyelitis without associated mediastinitis. Furthermore, the results of the ANN, in many aspects, not only reinforce the findings of the binary logistic model, but also provide other interesting insights into the phenomenon studied.

### Limitations

We recognize that this study has certain limitations. Firstly, its retrospective design raises the possibility of information bias. Furthermore, the fact that the present study was conducted at a single tertiary cardiac hospital that serves as a referral center for severe and complex cases suggests a relatively higher risk of death. These features might lead to variations in score performance when applied in hospitals with different clinical and microbiological profiles. Although the internal validation of the SWICS-30 score was satisfactory, the absence of a validation cohort warrants caution when interpreting our results. Further validation in other samples is necessary to ensure the reliability of our findings.

### CRediT authorship contribution statement

**Julio Alejandro Cedeno:** Conceptualization, Data curation, Formal analysis, Investigation, Methodology, Projectadministration, Resources, Writing – original draft, Writing – review & editing. **Tania Mara Varejão Strabelli:** Fundingacquisition, Writing – review & editing. **Bruno Adler Maccagnan Pinheiro Besen:** Software, Supervision, Validation,Visualization, Data curation. **Rafael de Freitas Souza:** Software, Supervision, Validation, Visualization, Data curation. **Denise Blini Sierra:** Data curation. **Leticia Rodrigues Goulart de Souza:** Data curation. **Samuel Terra Gallafrio:** Study design, Data curation. **Cely Saad Abboud:** Writing – review & editing. **Diego Feriani:** Writing – review & editing. **Rinaldo Focaccia Siciliano:** Funding acquisition, Conceptualization, Data curation, Formal analysis,Investigation, Methodology, Project administration, Resources, Writing – original draft, Writing – review & editing.

## Conflicts of interest

The authors declare no conflicts of interest.
